# Enabling accurate measurement of activated factor XI (FXIa) in therapeutic immunoglobulin products

**DOI:** 10.1111/vox.13046

**Published:** 2020-12-05

**Authors:** Helen V. Wilmot, Elaine Gray

**Affiliations:** ^1^ Haemostasis Section Biotherapeutics National Institute for Biological Standards and Control (NIBSC) South Mimms, Hertfordshire UK

**Keywords:** IVIG, immunoglobulins, plasma derivatives

## Abstract

**Background and objectives:**

In 2010, an intravenous immunoglobulin (IVIG) product was removed from the market due to an association with serious thromboembolic events. Investigations revealed that factor XIa (FXIa) was present as a process‐related impurity. This study investigated the ability of two commercial FXIa assays to measure FXIa in immunoglobulin preparations and conducted a survey of FXIa activity in marketed immunoglobulin products.

**Materials and methods:**

Factor XIa assays were modified to include spiking of samples with FXIa before testing. An immunoglobulin product and its excipient were used to assess the ability of the assays to recover the spiked FXIa levels.

**Results:**

The Biophen FXIa assay required a high pre‐dilution of the sample to obtain statistically valid results and complete FXIa recovery. The ROX FXIa assay was more sensitive, giving statistically valid results at a lower sample pre‐dilution and FXIa spike level. This modified ROX FXIa assay was used to assay 17 lots of immunoglobulin products for FXIa. Two product lots had measurable FXIa levels without the need for spiking. A further 3 lots produced detectable but not statistically valid FXIa results when left unspiked. Spiking produced statistically valid assays and recoveries above 100%, demonstrating inherent FXIa.

**Conclusion:**

This study shows marketed immunoglobulin products can contain detectable levels of FXIa. Spiking brings the FXIa levels into the quantifiable range of the assay, allowing measurement of inherent FXIa. Accurate measurement is important to inform on ‘safe’ levels of FXIa in these products and allow future safety guidelines to be set.

## Introduction

Human plasma‐derived immunoglobulin (IG) products are licensed to treat primary and secondary immunodeficiencies and conditions such as idiopathic thrombocytopenia purpura and Kawasaki disease. Off‐label use is also common for a wide range of conditions [1, 2]. Products are fractionated from human plasma pooled from thousands of healthy donors and contain mainly IgG antibodies. They can be given either as intravenous immunoglobulins (IVIG) or subcutaneous immunoglobulins (SCIG) and are generally safe and well tolerated; however, adverse events are numerous and range from mild (headaches, skin conditions) [3] to severe (renal impairment, thromboembolic events) [4, 5].

In 2010, the IVIG Octagam^®^ was temporarily removed from the market due to its association with a sudden increase in thromboembolic events (TE) [6]. This was followed by a withdrawal of the product Omr‐IgG‐am^®^ from the market and the association of the SCIG Vivaglobin^®^ with TE [7, 8]. Investigative work on Octagam^®^ identified the problem was the presence of a process‐related impurity, activated factor XI (FXIa), in the final product [9, 10]. A change in the manufacturing process improved the safety of the product [11] and the market suspension was lifted subject to additional testing being required [12]. Analysis of implicated lots of Octagam^®^ in a rabbit thrombogenicity model suggested that a FXIa content of ≥7 mU/ml FXIa was enough to cause thrombosis [9]; however, the Octagam^®^ study pre‐dated the establishment of an International Unit (IU) for FXIa in 2014 [13]. The unity of activity in the Octagam^®^ study is therefore not comparable to the IU.

In response to the adverse events, the European Pharmacopeia monographs for IVIG and SCIG were updated to state that ‘the method of preparation also includes a step or steps that have been shown to remove thrombosis‐generating agents’ [14, 15]. In order to show the absence or removal of procoagulant factors such as FXIa, accurate assay methods must be available. Methods such as the non‐activated partial thromboplastin time, thrombin generation assays and non‐specific FXIa chromogenic assays have been used for investigating procoagulant activity in IG products or their manufacturing intermediates [9, 16, 17, 18, 19]. At the time, specific FXIa chromogenic assays were not available, but two FXIa chromogenic assays are now commercially available and have been used by manufacturers to demonstrate the reduction or removal of FXIa during their manufacturing process [20, 21, 22]. However, lack of detection in an assay is not the same as showing an absence of activity, because it depends upon the limits of detection and quantification of the assays. Although there have not been reported increases in TE in IG products since the new monograph guidance has been introduced, there still needs to be an assessment of the suitability of the assays for determining procoagulant activity (or its absence) in IG preparations and some guidance on acceptable levels (if any) of FXIa may be helpful for manufacturers. This study therefore assesses the ability of both chromogenic kits to measure FXIa in IG products and uses marketed lots of various IG products to determine FXIa levels in products that have been used in patients.

## Methods

Assays were run on an ACL TOP 550 automated coagulometer (Werfen, Bedford, USA). Each sample was tested at a minimum of 3 dilutions, in duplicate, per assay.

### Development of spiking methods for FXIa quantification in IG samples

Factor XIa kits used in the study were the Biophen FXIa kit (Hyphen‐BioMed) and the ROX FXIa kit (Rossix AB) (both Quadratech, Cambridge, UK). Briefly, both kits rely on FXIa present in the sample to activate factor IX and subsequently factor X. Activated factor X cleaves a chromogenic substrate, causing a colour change that can be determined kinetically or by an end‐point method. This study employed the kinetic method.

Samples used for development of the spiking methods were IG‐A, a preparation of immunoglobulin (5% Flebogamma) freeze‐dried at NIBSC and its corresponding frozen excipient. Two marketed lots of 5% Flebogamma were used for proof of concept testing. The 1^st^ International Standard (IS) for FXIa, 13/100 (NIBSC, Potters Bar, UK) was used as the quantitative standard in the assays and for spiking experiments. FXIa spiking volume was incorporated into a minimum of a 1/2 pre‐dilution of the IG in kit buffer.

**Fig. 1:**
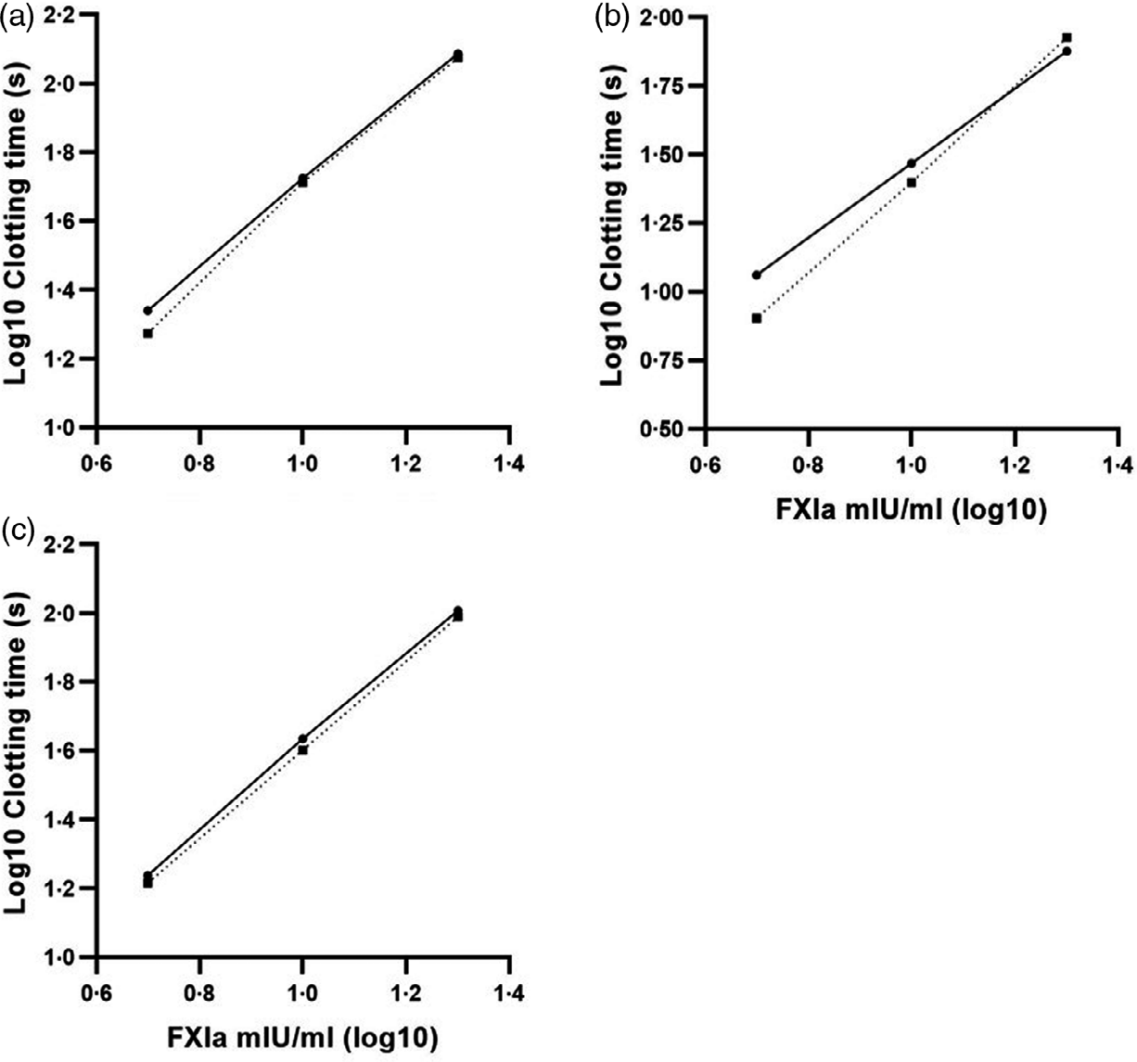
Example graphs plotting log_10_ clotting time (s) against log_10_ FXIa concentration for a test sample (dotted line) and FXIa standard (solid line). Parallelism was deemed acceptable when the ratio of the test slope to the standard as determined using Combistats^®^ was between 0.9 and 1.1 and the linearity was assessed visually from the graphs. The test sample is non‐linear in (A), linear but non‐parallel to the standard in (B), and linear and parallel to the standard in (C).

Parallel line analysis was used for calculation of activity estimates (CombiStats™ Version 6.0, Council of Europe). Equivalence testing in CombiStats^®^ with an acceptance criterion of 0·9–1·1 was used to compare the ratio of slopes between the standard and the sample dose–response to demonstrate parallelism. Linearity was assessed visually. Examples are shown in Fig. 1.

### Assay of marketed lots of IG products

The modified ROX FXIa method developed in this study was used to assay 17 lots of IG samples from 5 manufacturers (listed in Tables 3 and 4). All IVIG samples were initially tested with and without FXIa spiking at 1 mIU/ml. If the first assay of an unspiked product showed no dose–response (indicating no detectable FXIa), then the subsequent assays were performed on spiked samples only. SCIG products were also tested spiked and unspiked, but if unspiked samples produced a valid result against the standard, then spiking was deemed unnecessary. All spiked samples were pre‐diluted at least 1/2 during spiking. The minimum dilution necessary to achieve linearity and parallelism in the assay was used for each product and was usually 1/2, apart from Subgam^®^, which was assayed at pre‐dilutions of 1/4 or 1/6. Assays and analyses were carried out as described above, with three independent assays for each product. Some unspiked products showed a dose–response over 2 or 3 dilutions but were not parallel to the standard and, in some cases, were below the quantification limit of the assay. In these cases, the results are reported as 95% confidence limits (CL) only, to indicate that FXIa content was detectable but not accurately quantifiable.

A paired *t*‐test was used to compare differences between results, where appropriate.

## Results

### Development of methods to measure FXIa in immunoglobulin preparations – Biophen FXIa assay

Preparation IG‐A was loaded for assay at pre‐dilutions of 1/1, 1/2 and 1/4 in duplicate, followed by 1/1, 1/2, 1/4 and 1/8 dilutions within the assay on the analyser. No dose–response was observed in any of the assays (data not shown), and changes in absorbances were small, indicating that any FXIa present was below the level of quantification (LoQ). The IG‐A preparation (dilutions 1/2 to 1/35) and its corresponding excipient (dilutions 1/10 to 1/35) were then spiked with 20 mIU/ml purified FXIa to determine recovery. This pre‐dilution also allowed for the necessary volume of FXIa to be added to the IG sample and corresponds to the resulting dilution of the IG matrix. The results (Table 1) for both were assessed on 3 conditions: recovery of the expected levels of FXIa (taken as 95% confidence limits overlapping 20 mIU/ml), parallelism and linearity. A dilution of 1/35 (*n* = 3) was required to meet all three conditions for both the immunoglobulin preparation and its corresponding excipient. This suggests that a minimum of a 1/35 dilution allows for valid assay of immunoglobulins in the Biophen FXIa assay.

**Table 1:** Effect of sample pre‐dilution on the validity of the Biophen FXIa assay used to measure FXIa‐spiked immunoglobulin samples. Shaded boxes represent acceptable results for recovery of FXIa (95% confidence limits overlapping 20 mIU/ml), parallelism and linearity.

Sample and pre‐dilution	Recovery in mIU/ml (95% confidence limits)	Parallelism OK?	Linearity OK?
IG‐A 1/2	25.4 (22.3–28.9)	✗	✓
IG‐A 1/4	19.9 (17.5–22.7)	✗	✓
IG‐A 1/8	20.5 (18.3–22.9)	✗	✓
IG‐A 1/10	20.0 (17.3–23.2)	✓	✗
IG‐A Excipient 1/10	18.9 (16.7–21.5)	✓	✗
IG‐A 1/20	19.9 (17.2–23.1)	✗	✗
IG‐A Excipient 1/20	19.0 (16.7–21.5)	✓	✗
IG‐A 1/25	19.1 (17.8–20.4)	✓	✗
IG‐A Excipient 1/25	18.6 (17.3–20.0)	✓	✗
IG‐A 1/30	18.4 (17.5–19.5)	✓	✓
IG‐A Excipient 1/30	18.9 (18.3–19.5)	✓	✓
IG‐A 1/35	19.7 (18.8–20.7)	✓	✓
IG‐A Excipient 1/35	19.6 (18.7–20.5)	✓	✓
5% Flebogamma Lot 1 1/35	19.3 (17.7–20.9)	✓	✓
5% Flebogamma Lot 2 1/35	19.2 (18.2–20.2)	✓	✓

Based on a dilution of 1/35, this spiking method was used to test 2 marketed batches (Lot 1 and Lot 2) of 5% Flebogamma^®^, the same product type as IG‐A. In these assays (*n* = 3), there was acceptable FXIa recovery for the 2 lots (Table 1). All assays were valid for linearity and parallelism. This indicated that the 1/35 dilution worked well for therapeutic preparations of this product. No additional FXIa was recovered indicating that the assay did not detect inherent FXIa in these IG lots.

### Development of methods to measure FXIa in immunoglobulin preparations – ROX FXIa assay

Table 2 shows results from similar spiking experiments using the ROX FXIa assay. The kit insert suggests that the standard curve can range between 0·04 and 10 mIU/ml FXIa, with the option to use a narrower range of 0·04–1·4 mIU/ml if required. When the higher part of the standard curve was used, the responses for samples IG‐A spiked with 20 mIU/ml FXIa did not show acceptable parallelism or linearity. When a narrower range of the standard curve was used (0·04–0·31), the 20 mIU/ml spiked IG‐A samples required a dilution of 1/64 (a 1/16 on‐bench followed by doubling dilution from 1/4 on the analyser) to bring the FXIa levels into the range of the FXIa standard. This improved the statistical validity of the assays though the recovery was not quite 100%. When manufacturer lots of the same IG product were tested (5% Flebogamma^®^ Lots 1 and 2), the recovery for these was 100%. Recovery was very slightly lower than 100% for the spiked excipient (Table 2).

**Table 2:** Effect of sample pre‐dilution on the validity of the ROX FXIa assay used to measure FXIa‐spiked immunoglobulin samples. Shaded boxes represent acceptable results for FXIa recovery (95% confidence limits overlapping original spike levels), parallelism and linearity

Sample and pre‐dilution	High or narrow standard curve?	Assay dilutions	FXIa spike level (mIU/ml)	Recovery in mIU/ml (95% confidence limits)	Parallelism OK?	Linearity OK?
IG‐A 1/2.5	High	1/4–1/16	20	17.9 (17.5–18.3)	✓	✗
IG‐A Excipient 1/2.5	High	1/4–1/16	20	17.5 (17.1–17.8)	✓	✗
IG‐A 1/5	High	1/4–1/16	20	17.5 (17.2–17.9)	✓	✗
IG‐A Excipient 1/5	High	1/4–1/16	20	17.4 (17.1–17.8)	✓	✗
IG‐A 1/2	Narrow	1/16 then 1/4–1/16	20	18.9 (18.3–19.4)	✓	✓
IG‐A Excipient 1/2	Narrow	1/16 then 1/4–1/16	20	18.7 (18.2–19.3)	✓	✓
IG Lot 1 1/2	Narrow	1/16 then 1/4–1/16	20	19.8 (19.0–20.7)	✓	✓
IG Lot 2 1/2	Narrow	1/16 then 1/4–1/16	20	19.7 (19.0–20.6)	✓	✓
IG‐A Excipient 1/2	Narrow	1/16 then 1/4–1/16	20	18.9 (18.1–19.8)	✓	✓
IG‐A 1/2	Narrow	1/4–1/16	1	0.98 (0.95–1.02)	✓	✓
Excipient 1/2	Narrow	1/4–1/16	1	0.96 (0.84–1.09)	✓	✓
5% Flebogamma Lot 1 1/2	Narrow	1/4–1/16	1	0.99 (0.90–1.08)	✓	✓
5% Flebogamma Lot 2 1/2	Narrow	1/4–1/16	1	0.96 (0.87–1.07)	✓	✓

The use of the narrow standard curve for the Rossix FXIa assay meant that, at a spike level of 20 mIU/ml, a high dilution was required to bring the FXIa concentration into the range of the standard. A spike level of 1 mIU/ml was therefore investigated. At 1 mIU/ml, the analyser dilutions of 1/4 to 1/16 were sufficient to bring the FXIa content of the spiked sample into the range of the standard, without the need for additional dilutions beforehand. The results for IG‐A and its excipient (Table 2) showed that 100% recovery was obtained (*n* = 3) and the assays were valid for both parallelism and linearity. A 100% recovery with 95% CL overlapping 1 mIU/ml and valid assays (*n* = 3) were also obtained for the two 5% Flebogamma^®^ lots. No additional recovery of FXIa was observed, and neither IG lot had any measurable FXIa when tested unspiked, shown by a lack of dose–response across the dilutions (data not shown). By comparison with the 20 mIU/ml spike (95% CL approximately ± 1 mIU/ml), better sensitivity was obtained spiking with 1 mIU/ml, with 95% CL spanning approximately ± 0·1 mIU/ml of the measured value. The ROX FXIa assay using the 1 mIU/ml spiking method was therefore suitable for generating valid assays for therapeutic lots of IG and more likely to detect small amounts of inherent FXIa activity.

### Testing of marketed IG lots

Using the modified ROX FXIa assay with a spike level of 1 mIU/ml, 10 marketed IG products from 5 manufacturers (=17 lots) were evaluated for their FXIa content. Results are split into IVIG products (Table 3) and SCIG products (Table 4). The 5% Flebogamma^®^ results generated previously (Table 2) using the same method are replicated for comparison. Seven IVIG products (12 lots) were tested, and only the two lots of Vigam^®^ had detectable FXIa without spiking; however, due to non‐parallelism, the 95% CL only are reported. Spiking meant that valid assays were obtained and showed that the recovery after spiking increased by an amount corresponding to the 95% CL of the unspiked product. All IVIG samples were tested with a pre‐dilution of 1/2 to allow for spiking. The additional recovery of 0·30 and 0·26 mIU/ml FXIa for Vigam^®^ lots 1 and 2, respectively, translates to 0·60 and 0·52 mIU/ml FXIa in the undiluted product. All other products had a recovery of 100% of FXIa (taken as 95% confidence limits overlapping 1 mIU/ml), apart from Privigen^®^ and one lot of 10% Flebogamma^®^, which both had recovery slightly less than 100%. The assays were all statistically valid, so it is possible that there was a small amount of inhibitory activity in these samples.

**Table 3:** Measurement of FXIa in marketed intravenous immunoglobulin samples using the modified ROX FXIa assay.

Manufacturer	Product (IVIG)	Pre‐dilution	FXIa level in mIU/ml (95% confidence limits)		
			Unspiked sample	Sample spiked with 1 mIU/ml FXIa	Estimated in product
BPL	Gammaplex Lot 1	1/2	<LOQ	1.02 (0.88–1.19)	‐
	Gammaplex Lot 2	1/2	<LOQ	1.00 (0.98–1.02)	‐
	Vigam Lot 1	1/2	NP (0.26–0.31)	1.30 (1.29–1.31)	0.60 (0.58–0.62)
	Vigam Lot 2	1/2	NP (0.23–0.34)	1.26 (1.21–1.31)	0.52 (0.42–0.62)
CSL Behring	Privigen	1/2	<LOQ	0.94 (0.94–0.95)	‐
Grifols	5% Flebogamma Lot 1	1/2	<LOQ	0.99 (0.90–1.08)	‐
	5% Flebogamma Lot 2	1/2	<LOQ	0.96 (0.87–1.07)	‐
	Gamunex	1/2	<LOQ	0.96 (0.92–1.00)	‐
	10% Flebogamma Lot 1	1/2	<LOQ	0.93 (0.82–1.05)	‐
	10% Flebogamma Lot 2	1/2	<LOQ	0.94 (0.91–0.97)	‐
Kedrion	IG Vena Lot 1	1/2	<LOQ	0.97 (0.91–1.04)	‐
	IG Vena Lot 2	1/2	<LOQ	1.00 (0.98–1.01)	‐

NP, non‐parallel; <LOQ, below the limit of quantification

**Table 4:** Measurement of FXIa in marketed subcutaneous immunoglobulin samples using the modified ROX FXIa assay.

Manufacturer	Product (subcutaneous immunoglobulin)	Pre‐dilution	FXIa level in mIU/ml (95% confidence limits)		
			Unspiked sample	Sample spiked with 1 mIU/ml FXIa	Estimated in product
BPL	Subgam Lot 1	Accounted for within results	4.13 (3.32–5.22)	Not required	4.13 (3.32–5.22)
	Subgam Lot 2	Accounted for within results	1.53 (1.45–1.62)	Not required	1.53 (1.45–1.62)
CSL Behring	Hizentra	1/2	<LOQ	0.94 (0.90–0.97)	‐
Octapharma	Gammanorm Lot 1	1/2	<LOQ but ~0.1 (0.11–0.15)	1.08 (0.96–1.21)	‐
	Gammanorm Lot 2	1/2	NP (0.16–0.37)	1.18 (1.05–1.33)	0.36 (0.1–0.66)

NP, non‐parallel; <LOQ, below the limit of quantification.

Three different SCIG products from 3 different manufacturers (5 lots in total) were tested (Table 4) using the same method as for the IVIGs, except that one product (Subgam^®^) was found not to require spiking with FXIa. Both lots of this product had enough inherent FXIa (approximately 4 and 1·5 mIU/ml) such that the dilution required to bring the FXIa levels into the range of the standard was sufficient to remove any matrix effect and produce valid assays. This dilution has been accounted for in the data. Hizentra^®^ showed no additional FXIa activity when spiked, and the less than 100% recovery suggests some FXIa inhibitory activity in the sample. The 2 lots of unspiked Gammanorm^®^ had low levels of FXIa (0·1 and 0·4 mIU/ml for Lots 1 and 2, respectively), but assays were non‐parallel. When spiked, recovery of Lot 1 was 1·1 mIU/ml, but with 95% CL overlapping 1 mIU/ml, this increase in recovery was not significant. For Lot 2, the spiked sample showed a corresponding increase in recovery above 1 mIU/ml FXIa, without 95% CL overlapping 1 mIU/ml. Taking into account the 1/2 pre‐dilution, this equated to a FXIa level in the product of around 0·4 mIU/ml. This demonstrates that the modified ROX FXIa assay is sensitive to very small levels of inherent FXIa in IG samples.

### Retesting of Subgam^®^

During the testing of the Subgam^®^ Lot 1 in the ROX FXIa assay, it was noted that the FXIa activity increased over time. After initial assays, activity was measured again 5 and 8 months later. The results (Table 5) showed that initial FXIa levels of around 4·2 mIU/ml (as reported in Table 4) increased to 5·7 and 7·1 mIU/ml, respectively, after 5 and 8 months. The difference in activity was significant (*P* = 0·034) between 0 and 5 months and between 0 and 8 months (*P* = 0·016). The 95% CL for the 5‐ and 8‐month testing overlapped, with no statistical difference between the results (*P* = 0·115). This indicated that the FXIa activity increased over time but then plateaued. The two lots of IG with the next highest FXIa activity, Subgam^®^ Lot 2 and Vigam^®^ Lot 1, were also retested for any change in FXIa activity after 8 months. Subgam^®^ Lot 2 showed a significant (*P* = 0·02) increase from 1·53 mIU/ml (Table 4) to 3·18 mIU/ml (95% CL 2·65–3·82) after 8 months, and spiked samples of Vigam^®^ Lot 1 showed a slight decrease in activity from 1·30 mIU/ml, to 1·17 mIU/ml (95% CL of 1·06–1·28; *P* = 0·04) after 8 months. Testing of Gammanorm^®^ Lot 2 after 3 months showed no change in activity, at 1·19 mIU/ml (95% confidence limits of 1·16–1·23; *P* > 0·05). The cause of the changes in activity over time is unknown, however could be due to autoactivation of FXI in the product to FXIa, and decreases in activity are most likely due to degradation of FXIa.

**Table 5:** Testing of Subgam Lot 1 in the ROX FXIa assay (unspiked) over time, and in the Biophen FXIa assay (unspiked and spiked with 20 mIU/ml FXIa).

Assay type	Spike level (mIU/ml)	Subgam Lot 1 FXIa level in mIU/ml (95% confidence limits)
ROX time 0 months	Not required	4.16 (3.31–5.22)
ROX + 5 months	Not required	6.57 (5.67–7.61)
ROX + 8 months	Not required	7.08 (6.54–7.66)
Biophen + 8 months	None	NP (6.48–8.80)
Biophen + 8 months	20	19.1 (18.2–20.1)

NP, non‐parallel.

With Subgam^®^ lot 1 having an increased activity of 7 mIU/ml, there was a possibility that the activity could be detected using the Biophen assay. A sample was therefore tested unspiked at a pre‐dilution of 1/2 in the assay, and also using a 1/35 dilution spiked with 20 mIU/ml FXIa, as recommended earlier. The results showed that there was a dose–response for the unspiked Subgam^®^ in the assay, however only for the first two dilutions. The response was also not parallel to the standard. The 95% CL estimated the FXIa content to be 6·5–8·8 mIU/ml (Table 5). This is in agreement with the ROX FXIa assay performed at the same time (7·1 mIU/ml), however cannot be considered to be accurate because of the non‐validity of the results. The spiked sample, though producing valid results, did not show any increase in recovery of spiked FXIa, since the 95% CL overlapped 20 mIU/ml (Table 5). This shows that, owing to the high dilution needed, the modified Biophen assay was not sensitive enough to measure FXIa in this sample, despite it having a relatively high content.

## Conclusions

This study has developed methods for valid measurement of FXIa in IG products. Both commercially available FXIa chromogenic kits, ROX and Biophen, were investigated. The Biophen assay has a detection limit of ~2·5 mIU/ml, according to the package insert [23]. Using parallel line assay with a minimum of 3 doubling dilutions, with 2·5 mIU/ml being the lowest concentration tested, equates to a suggested limit of quantification (LOQ) of 10 mIU/ml. In practice, when assaying the spiked IG samples, the dilution corresponding to 2·5 mIU/ml was often not linear, even for the standard. Loading the appropriately diluted samples with a spike level of 20 mIU/ml allowed for testing at 20, 10 and 5 mIU/ml (1/1, 1/2 and 1/4) and gave statistically valid assays, once matrix interference had been removed. Development work with 5% Flebogamma^®^ samples suggested that a pre‐dilution of 1/35 removed any matrix interference and allowed statistically valid assays to be obtained. Therefore, for measurement of FXIa in spiked IG samples, we have found the Biophen FXIa assay to have an LOQ of 20 mIU/ml using parallel line assay, with a suggested pre‐spike dilution of 1/35.

The ROX FXIa states the single‐point limit of detection to be 0·03 mIU/ml [24], which would equate to a quantification limit of around 0·12 mIU/ml by parallel line assay using 3 doubling dilutions. In this study, the LoQ using parallel line assay was 0·25 mIU/ml FXIa, based on 3 doubling dilutions corresponding to concentrations of 0·25, 0·125 and 0·0625 mIU/ml. The next doubling dilution (0·03125 mIU/ml), though detectable and showing a dose–response, often led to some non‐linearity. With the increased sensitivity, a lower spike level of 1 mIU/ml FXIa could be used, and a lower overall dilution was required to remove matrix interference. For all spiked samples, a pre‐dilution of 1/2 followed by in‐assay dilutions of 1/4 onwards was sufficient to obtain statistically valid assays. For the samples that had a higher level of inherent FXIa (>1 mIU/ml), no spiking was required, and the pre‐dilution could be adjusted to bring the levels into the range of the standard without interference from the sample matrix.

Overall, the spiking of immunoglobulin with FXIa reduced the effect of the matrix on the linearity and parallelism of both assays and allowed statistically valid assays to be obtained. The ROX assay had a higher sensitivity and was therefore chosen for measurement of the marketed IG products. Of the 10 products studied, only 3 (Vigam^®^, Subgam^®^ and Gammanorm^®^) had detectable levels of FXIa. Without the spiking modification to the assay, only Subgam^®^ could have validly been measured by the ROX FXIa assay using parallel line analysis. To our knowledge, this is the first study that has reported measurable levels of FXIa in identified IG products. The manufacturer of Vigam^®^ has ceased production of the product; however, vials may still be being used until the expiry date (usually 3 years from manufacture date) has passed. Vigam^®^ is an IVIG product, and no patient safety issues have been raised, therefore is it reasonable to assume that the low levels of FXIa measured in these lots have not caused excess adverse effects in patients. The other two products with measurable FXIa were SCIG products. It is thought that the increase in serum viscosity after administration makes TE more likely to occur with IVIG products [25]. Therefore, despite the presence of FXIa, the subcutaneous administration route of Subgam^®^ and Gammanorm^®^ makes TE less likely. Patient safety may therefore be uncompromised by higher levels of FXIa in SCIG compared to IVIG products.

In this study, the levels of FXIa in the Subgam^®^ lots increased over time. An increase in FXIa in IG preparations upon storage has been observed previously [26] and is most likely due to the ability of FXIa to autoactivate FXI [27]. Once all FXI in the product has been activated, it is likely that the FXIa levels will decrease as the FXIa degrades. This may explain the reduction in activity in Vigam^®^ Lot 1 observed in this study.

Despite the relatively high amount of FXIa present in Subgam^®^ Lot 1, the Biophen assay was unable to detect this when the spiking method was used. This is because high dilutions of IG samples are required to produce valid results in this assay. Caution should therefore be used if using this assay to assess FXIa in IG samples, since lack of a positive result could still mean the content is as high as 7 mIU/ml or more. When Subgam^®^ Lot 1 was tested unspiked, only the two lowest dilutions showed a dose–response and the results were not statistically valid due to non‐parallelism with the standard. The 95% CL suggested a value of 6·5–8·8 mIU/ml. If single‐point analysis was used, the results would have been 7·3 and 8·8 mIU/ml, respectively, for the 1/2 and 1/4 total dilutions of the product. Whilst these values agree reasonably well with the FXIa content as measured using the ROX FXIa assay (7·1 mIU/ml), the lack of assay validity by parallel line assay means the results cannot be considered accurate. Use of single‐point analysis only would not have indicated that the assay results were statistically invalid and demonstrates the importance of using multiple dose–responses with parallel line analysis. The detection of FXIa in the unspiked sample suggests that this assay could be used to screen IG preparations for high levels of FXIa (7 mIU/ml or more), but it may not be suitable for accurate determination of FXIa levels. However, spiking FXIa in the samples at different levels may help to improve the sensitivity and validity of this assay.

There is a European Pharmacopeial requirement for IG manufacturers to demonstrate the reduction or removal of procoagulant activity from their products [14, 15], and therefore, sensitive and accurate assays are required. This study has shown that both the ROX and Biophen FXIa chromogenic kits can be used to validly assay FXIa content in IG preparations, with the ROX kit having greater sensitivity. The ability to measure low levels of FXIa in products may assist manufacturers in demonstrating the reduction of FXIa in their products, as required by the relevant monographs [14, 15], even though the measured levels may be below that associated with adverse events. We recommend that measurement of FXIa in IG preparations should be optimized within each laboratory, since suitable conditions may vary according to whether the assays are run manually or using a coagulometer, the samples being tested, and the method of data collection (kinetic or end‐point). Assays should use multiple dilutions and attention should be paid to linearity and parallelism of the test responses relative to the standard. Spiking of FXIa should be used in the case of samples that do not exhibit a dose–response when tested unspiked, or where the response is not parallel to the standard.

The European Pharmacopoeia is considering the introduction of a test for FXIa content in final IG products and the suitability of the commerical kits may depend on the specifications set. By assaying marketed lots of 10 different IG products, this study has informed on the FXIa content that may be considered safe, since no additional adverse effects have been associated with their use. For SCIG, the highest level observed during initial testing was 4·2 mIU/ml FXIa, and 0·6 mIU/ml FXIa for IVIG. The increase in FXIa content in Subgam^®^ over time indicates that there may be a need to monitor these products during the shelf life of IG products. None of the IG lot numbers used in this study are known to be associated with adverse events; however, the observations made in this study may be useful in setting future guidelines and acceptable limits for FXIa content in these medically important products.

## Conflict of interests

The authors have no conflict of interests. No funding was received for this study.
